# When Coaching Meets Mentoring: Impact of Incorporating Coaching into an Existing Mentoring Program at a Community Hospital

**DOI:** 10.7759/cureus.3138

**Published:** 2018-08-14

**Authors:** Radhika Kakarala, Susan J Smith, Esteban Barreto, Karen Donelan, Kerri Palamara

**Affiliations:** 1 Internal Medicine, Mclaren Flint, Flint, USA; 2 Medicine, Mclaren Flint, Flint, USA; 3 Medicine, Massachusetts General Hospital, Boston, USA

**Keywords:** resident coaching program, mentoring, physician burnout, positive psychology coaching, faculty coaches, emotional exhaustion, resident performance, resiliency, mindful listening, self-reflection

## Abstract

Introduction

One in two physicians experiences professional burnout. Resident coaching is a novel method to provide emotional support and professional development to residents. The feasibility of implementing coaching at a community hospital has not been reported. This curricular innovation examined the feasibility and impact of integrating positive psychology coaching at a community hospital.

Methods

The Massachusetts General Hospital (MGH) Professional Development Coaching Program (PDCP) curriculum was used to train faculty coaches and for the informal pre-coaching session refreshers. Participants were paired and expected to participate in four 1:1 coaching sessions. The impact of the PDCP was assessed through pre- and post-PDCP online surveys.

Results

Twelve interns and nine faculty coaches were included in the program and surveyed. Survey completion was 10/12 (83%) and 6/9 (67%) at baseline and 9/12 (75%) and 7/9 (78%) at end of year (EOY) for interns and coaches, respectively. For interns, Emotional Exhaustion (EE) using the Maslach Burnout Inventory (MBI) was high or medium for 60% of respondents at baseline, and 56% at EOY. Fifty percent of coaches scored medium on EE at baseline, compared to only 14.3% at EOY. Seventy-five percent of respondents rated their PDCP experience as excellent or good. Nearly all interns rated the quality of communication with their coaches highly on a five-point Likert Scale.

Conclusions

Implementation of a coaching program in a community hospital residency program is feasible. Burnout using the MBI was stable from beginning to EOY for interns, but improved for coaches. Interns and coaches rated their professional development coaching program experience highly, and would recommend it to others.

## Introduction

One in two physicians experience burnout during their career, across all specialities [1]. Burnout is described as a “prolonged response to chronic stressors on the job, and is defined by exhaustion, cynicism, and inefficacy” [2]. Common factors associated with burnout include younger age [3], time constraints, lack of control over work schedule [4], self-imposed perfectionism and guilt, administrative burden, poor self-care [5], and lack of self-awareness [2,3]. Conversely, engagement in work, characterized by energy, involvement, and efficacy, is thought to be protective against burnout [2].

Physician coaching using a positive psychology approach is a novel method to help prevent and address physician burnout and enhance engagement [6,7]. The Massachusetts General Hospital (MGH) Professional Development Coaching Program (PDCP) was designed to optimize resident performance through principles of positive psychology [8], using a strength-based model. This model has been replicated at other large university programs, but the feasibility and impact of implementing coaching into a small community program has not been explored. This article reports the feasibility and impact of implementing a coaching program at a community hospital. The intent of this program was to decrease the interns’ burnout and increase their resiliency by allowing them to: 1) visualize their best selves, 2) set realistic goals and strategies, and 3) become mindful of their accomplishments.

## Materials and methods

The community-based categorical internal medicine program of McLaren Flint hospital is composed of a diverse group of mostly foreign medical graduate faculty and residents. There are 12 residents in each year’s cohort. The program also retains nine full-time and one part-time faculty. The previous approach to mentoring in the residency program utilized a combination of academic advisors, peer and faculty mentoring. Following the MGH model, we implemented the PDCP as a pilot, initially targeting interns. All of the 12 interns were non-US international medical graduates; there were four females. They were informed of the additional support that they would receive from a faculty coach and how that role differed from their resident mentor’s role.

Our residency program supported and assisted a faculty member in becoming a certified Health and Wellness Coach and a Coaching Program Director (CPD). The CPD attended a national precourse taught by MGH’s CPD, where they described their model and provided tools for participants to replicate this program at other institutions. Our CPD then collaborated with the MGH CPD to implement the model within this residency program.

Coach training and assignment

Nine core faculty were invited to participate as a coach and underwent two hours of training facilitated by the CPD. Coaches were provided MGH PDCP guides for the four coaching sessions. The training combined theory and real-life practice utilizing positive psychology, mindful listening, self-reflection, exploration of possible solutions, and validation of the coachee’s self-identified strategies to reach goals. The principles embedded in our positive psychology approach are shown in Table 1. The coaching principles and curriculum draw extensively from the positive psychology and coaching literature; our specific materials were developed by Dr. Carol Kaufman and Dr. Kerri Palamara McGrath. The full curriculum and materials were published in a study by Palamara et al. [6].

**Table 1:** Positive psychology coaching principles.

Begin each session asking for their happiest and most satisfying recent experiences, either professional or personal
Conscious shift of the resident’s focus from what they are struggling with, to their successful experiences and building upon that
Conscious focus on their success stories, and encouraging them to journal about their experiences of making a difference in people’s lives
Connection with strengths and how to use those in new or different ways to overcome challenges
Begin year with painting the picture for the “perfect” intern year to encourage dreaming broadly, revisit this image each meeting to self-assess, reframe goals, and raise their bar higher
Set short-term goals based on upcoming challenges as well as baby-steps toward the big-picture goal

Interns and faculty were each matched sequentially by pseudorandomization (alphabetical order). Due to the importance of the resident being able to share weaknesses and vulnerabilities with their coaches we were concerned about coach-coachee relationships in which the coach might, at some time, evaluate the trainee. The fear was that residents would be unwilling to identify their weaknesses and vulnerabilities to someone who might later evaluate them. However, because of the small size of our faculty and their multiple teaching and supervising roles, we were unable to avoid potential coach-coachee and evaluator-resident relationships.

Assessment

Interns and coaches were surveyed at baseline before implementation of the program in September 2015 and at end of year (EOY) in May 2016. The outcome measures collected in this evaluation included: 1) experience with the program; 2) professional development goals and activities; 3) professional interactions and working relationships with colleagues; 4) assessment of personal accomplishment (PA) and emotional exhaustion (EE) as measured by the Maslach Burnout Inventory (MBI). MBI is widely used to measure burnout [2]. For this study, we used two subscales of the full inventory developed for medical personnel. We measured emotional exhaustion and personal accomplishment.

Our institutional review board exempted this study.

## Results

At baseline, survey response rate was 83% (10/12) for interns and 67% (6/9) for coaches. At EOY, survey response rate was 75% (9/12) for interns and 78% (7/9) for coaches. As shown in Table 2, PA score was high or medium for both interns and coaches at both baseline and EOY. Interns scored high or medium on EE score 60% (6/10) at baseline, compared to 55.5% (5/9) at EOY. None of the coaches had high burnout. Half of the coaches (3/6) scored medium on EE at baseline, compared to only 14.3% (1/7) at EOY. After one year of the PDCP, 78% of interns (7/9) and 86% of coaches (6/7) rated their coaching experience as excellent or good. Nearly 90% of interns (8/9) rated the quality of their communication with their coach as excellent or good. Most interns (89% of interns [8/9]) and faculty (71% [5/7]) definitely or probably would advise other residency programs to implement a PDCP.

**Table 2:** Coaching program experience.

	Baseline Intern	Follow-up Intern	Baseline Coach	Follow-up Coach
	N = 10 (%)	N = 9 (%)	N = 6 (%)	N = 7 (%)
Maslach Burnout Inventory				
Personal Accomplishment				
High	6 (60.0%)	3 (33.3%)	3 (50.0%)	3 (42.9%)
Medium	4 (40.0%)	6 (66.6%)	2 (33.3%)	3 (42.9%)
Low	0 (0%)	0 (0%)	1 (16.6%)	1 (14.3%)
Emotional Exhaustion				
High	2 (20.0%)	2 (22.2%)	0 (0%)	0 (0%)
Medium	4 (40.0%)	3 (33.3%)	3 (50.0%)	1 (14.3%)
Low	4 (40.0%)	4 (44.4%)	3 (50.0%)	6 (85.7%)
Experience in the Coaching Program				
Excellent	x	3 (33.3%)	x	4 (57.1%)
Good	x	4 (44.4%)	x	2 (28.6%)
Fair/Poor	x	1 (11.1%)	x	1 (14.3%)
Quality of Communication with Coach				
Excellent	x	5 (55.5%)	x	x
Good	x	3 (33.3%)	x	x
Fair/Poor	x	0 (0%)	x	x
Recommend Coaching to Future Programs				
Definitely Would	x	5 (55.5%)	x	4 (57.1%)
Probably Would	x	3 (33.3%)	x	1 (14.3%)
Definitely/Probably Would not	x	0 (0%)	x	2 (28.6%)

We asked interns how they were using skills they learned in the coaching program. Interns reported using coaching skills in their interactions with colleagues in medicine (77.8%) and nursing (66.7%), with patients (77.8%), with family and friends (55.5%) and with mentors and advisors (66.6%). When asked, “What is one aspect of the program that they would keep,” interns indicated they would like to retain meetings with both academic advisors and coaches and receive coaching on work-life balance.

## Discussion

This investigation explored how a coaching program validated at a university academic hospital could be piloted and integrated into a community hospital residency program, primarily containing international medical graduates. Nearly all interns rated the quality of communication with their coaches highly. Their burnout was slightly below national averages. Despite the differences in program size and composition, interns reported comparable satisfaction as that of the MGH interns with the PDCP and were willing to recommend it to other residencies, indicating that the positive psychology-based coaching was helpful to their progress.

We included only core program faculty members as coaches for ease of coordinating coach training and their accessibility to interns. The use of the MGH PDCP curricular material [6], and access to the expertise of their CPD, combined with our own certified health and wellness CPD, were key factors to the successful implementation of this program. In addition, the cultural similarities between the residents and most of the faculty may have played a role in the increased resident satisfaction about the quality of communication with their coaches. Interestingly, while the interns’ emotional exhaustion score remained the same from baseline to EOY, the coaches had an improvement in their EE score. Our hypotheses explaining these differences are that while interactions between coaches and coachees using positive coaching techniques had highlighted the interns’ tremendous growth in competence in their patient care and the successes they had achieved over the year, the interns were also facing the stressful situation of being promoted to a job that they had not yet done, one involving more responsibility for patients’ lives while receiving less supervision. In addition, they were faced with the responsibility of training an intern and may have experienced anxiety over their teaching and supervision skills. On the other hand, the faculty coaches could breathe a sigh of relief. They knew based on their cumulative experience that the interns were ready to handle the responsibility of PGY 2 at the end of their internship, even though the interns did not have the wisdom of that hindsight.

It has been shown that the learning environment strongly depends on faculty well-being [9], and this research positions future studies for exploring this further in the context of faculty involvement in a coaching program. Given no change in interns’ EE and the Accreditation Council for Graduate Medical Education’s mandate to improve physician well-being [10-12], we added a question about their professional and personal wellness in every coaching session, since strategies that are specific to an individual can lead to reductions in the physician burnout [13]. The intent of this inquiry was to help residents reflect on how they are handling the physical and emotional exhaustion of residency by asking them “How are you dealing with the stress of residency? What are you doing to take care of yourself?” This was also to facilitate discussion about their self-care behaviors.

Limitations of our study are that it was conducted at a single community internal medicine residency program with a small sample size limited to interns. In addition, we were unable to exclude faculty who would serve in an evaluative role. However, our successful implementation and resident satisfaction with their coaches in a small, community-based program would suggest that other programs might be able to implement this PDCP regardless of their size and faculty roles.

## Conclusions

We found that it is feasible to implement a professional development coaching program used at a university hospital in a community hospital residency program. Both the interns as well as the coaches involved in this found it to be beneficial. Future studies evaluating the impact of positive psychology-based PDCPs with a focus on residents’ personal well-being and its impact on their emotional exhaustion will be helpful.
